# Brain Structural Alterations, Diabetes Biomarkers, and Cognitive Performance in Older Adults With Dysglycemia

**DOI:** 10.3389/fneur.2021.766216

**Published:** 2021-10-28

**Authors:** Dae Jong Oh, Ji-Jung Jung, Seong A. Shin, Hairin Kim, Soowon Park, Bo Kyung Sohn, Bo Kyung Koo, Min Kyong Moon, Yu Kyeong Kim, Jun-Young Lee

**Affiliations:** ^1^Department of Psychiatry, Seoul National University College of Medicine, Seoul, South Korea; ^2^Department of Psychiatry, SMG-SNU Boramae Medical Center, Seoul, South Korea; ^3^Department of Surgery, Seoul National University Bundang Hospital, Seongnam, South Korea; ^4^Department of Nuclear Medicine, SMG-SNU Boramae Medical Center, Seoul, South Korea; ^5^Division of Teacher Education, College of General Education for Truth, Sincerity and Love, Kyonggi University, Suwon, South Korea; ^6^Department of Psychiatry, Inje University Sanggye Paik Hospital, Seoul, South Korea; ^7^Department of Internal Medicine, Seoul National University College of Medicine, Seoul, South Korea; ^8^Department of Internal Medicine, SMG-SNU Boramae Medical Center, Seoul, South Korea; ^9^Department of Psychiatry and Neuroscience Research Institute, Seoul Nation University College of Medicine, Seoul, South Korea

**Keywords:** diabetes, prediabetes, dysglycemia, brain structure, white matter integrity, cognition

## Abstract

Despite the high risk of dementia in older adults with type 2 diabetes, the neuroanatomical correlates of cognitive dysfunction that are particularly affected by diabetes are not well characterized. This study is aimed to examine the structural brain alterations in dysglycemic older adults. Using voxel-based morphometric and tract-based spatial statistics, we examined changes in gray matter volume, white matter volume, and microstructural integrity in older adults with prediabetes and diabetes. We also assessed the correlation of these structural changes with diabetes biomarkers and cognitive performance. A total of 74 non-demented older adults (normal, *n* = 14; prediabetes, *n* = 37; and diabetes, *n* = 23) participated in this study and underwent structural and diffusion magnetic resonance imaging (MRI) scans and neuropsychological tests. Subjects with diabetes showed reduced volume of cerebellar gray matter and frontal white matter and diffuse white matter dysintegrity, while those with prediabetes only showed reduced volume of insular gray matter. Atrophic changes in the cerebellum and frontal lobe and frontal white matter dysintegrity were correlated with chronic hyperglycemia and insulin resistance and worse performance in verbal memory recognition and executive function tests. Our findings suggest that chronic hyperglycemia and insulin resistance may alter brain structures forming the fronto-cerebellar network, which may cause cognitive dysfunction in older adults.

## Introduction

The prevalence rates of both type 2 diabetes and dementia increase with age, and older adults are often concomitantly affected by both diseases (1). Previous studies have found that type 2 diabetes increases the risk of cognitive decline and incident dementia (2–4). However, the neuroanatomical correlates that can be affected by diabetes-related pathophysiology and finally lead to neurodegeneration are not well characterized.

Many researchers have reported alterations of brain structures in diabetes; however, there are several unresolved questions. It remains unclear whether volume changes in specific brain areas are particularly associated with diabetes. With the exception of a few studies that estimated the regional distribution of brain atrophy (5–8), most previous studies have only focused on the volumes of total brain, total gray matter, total white matter, and hippocampus in diabetes (9–22). The lack of voxel-based morphometry (VBM) studies in this field has limited the identification of unexpected regions related to the pathophysiology of diabetes. Furthermore, it has not been identified whether alteration of brain atrophy in specific regions is correlated with cognitive decline in dysglycemic older adults. Finally, as most prior studies did not include a separate prediabetes group, the structural brain alterations in the early stage of dysglycemia are not clear. Although several studies compared brain volumes of prediabetes with normal control, none of these studies (8, 15, 17, 20), with the exception of the Maastricht study (22), found any significant difference in this respect. Use of diffusion tensor imaging (DTI) or VBM analysis may help overcome the limitations of previous studies; however, these modalities have not been applied to participants with prediabetes.

In the present study, using structural magnetic resonance imaging (MRI), we applied voxel-based morphometry to localize specific and unexpected regions with reduced gray and white matter volume and tract-based spatial statistics to identify regions with microstructural alterations in older adults with dysglycemia, including prediabetes and diabetes. We also assessed whether the volume of specific brain regions is affected by diabetes biomarkers, and whether these regions are associated with poor cognitive performance.

## Materials and Methods

### Study Design and Participants

In this cross-sectional study, we recruited 74 participants (age range: 52–85 years) from the Dementia and Memory Disorder Clinic at the SMG-SNU Boramae Medical Center (Seoul, Korea) and via advertisements. The clinical characteristics (age, sex, education, dementia status) and laboratory data (plasma glycosylated hemoglobin, HbA1c; fasting plasma glucose, FPG; Homeostatic Model Assessment for Insulin Resistance, HOMA-IR) were obtained by reviewing medical records. The exclusion criteria included dementia, structural lesions on brain imaging, major neurological or physical illness that may affect cognitive performance, history of alcohol or drug abuse in the preceding ten years, presence of visual or hearing difficulties or motor impairment, and inadequate or uncooperative attitude during the test.

This study was approved by the Institutional Review Board of the Seoul National University Hospital, and written informed consent was obtained from all participants.

### Assessment for Diabetes and Related Biomarkers

All participants fasted for over 14 h prior to venous blood sampling for laboratory testing. On the basis of FPG, HbA1c, and previous medical history, the participants were classified into the following three groups based on the 2016 revision of the diagnostic criteria for diabetes by American Diabetes Association (23): type 2 diabetes group (*n* = 23) with HbA1c ≥ 6.5% or FPG ≥ 126 mg/dL; prediabetes group (*n* = 37) with HbA1c 5.7–6.4% or FPG 100–125 mg/dL; and normal control group with normal glucose tolerance (NL; *n* = 14) with HbA1c < 5.7% and FPG <100 mg/dL. Dysglycemia was defined as either having prediabetes or diabetes according to the above cutoffs.

Insulin resistance according to the Homeostatic model assessment of insulin resistance (HOMA-IR) was calculated using the following formula: HOMA-IR = fasting plasma insulin (U/mL) × FPG (mg/dL)/405 (24).

### Assessment of Cognitive Performance

Geriatric psychiatrists administered a standardized diagnostic interview with neurological and physical examinations to all participants using the Korean version of the Consortium to Establish a Registry for Alzheimer's Disease (CERAD-K) Assessment Packet Clinical Assessment Battery (25). Professional neuropsychologists administered the CERAD-K neuropsychological battery tests to all participants (26). CERAD-K neuropsychological battery was composed of the following subtests: word list memory test, word list recall test, word list recognition test, constructional recall test, constructional praxis test, verbal fluency test, trail making test (A and B), and modified Boston naming test. The diagnostic panel consisting of geriatric psychiatrists and neuropsychologists confirmed the diagnosis of dementia, and participants diagnosed as having dementia were excluded from the current study.

### Brain Image Acquisition and Preprocessing Procedures

Brain structural T1-weighted and DTI sequences were obtained using a 3.0 Tesla MRI scanner (Achieva, Philips Healthcare, Netherlands). The acquisition parameters for structural T1-weighted image were as follows: repetition time, 9.9 ms; echo time, 4.6 ms; slice thickness, 1; image size, 224 × 224 × 180; voxel size, 1.00 × 0.98 × 0.98 mm. The protocols for DTI were as follows: number of diffusion gradient directions, 32 [one b0 image and 31 diffusion-weighted images (*b* = 1000 s/mm2)]; slice thickness, 2 mm; repetition time, 8,097 ms; echo time, 68 ms; flip angle, 90°; image size, 144 × 144 × 70; voxel size, 1.53 × 1.53 × 2.00 mm.

The structural T1-weighted image preprocessing steps for VBM analysis were performed using Statistical Parametric Mapping 12 (SPM12, Wellcome Department of Imaging Neuroscience, UCL, UK, https://www.fil.ion.ucl.ac.uk/spm/software/spm12/) and Computational Anatomy Toolbox (CAT12, http://dbm.neuro.uni-jena.de/cat) implemented in Matlab [(27), http://www.mathworks.com]. Initially, structural T1-weighted images were corrected for inhomogeneity and were noise- and skull-stripped. Gray matter and white matter were then segmented and normalized into a standard space using DARTEL (diffeomorphic anatomical registration using exponentiated lie algebra) algorithms and tissue probability maps. The images were modulated to preserve tissue volume after warping and finally smoothed with an isotropic Gaussian kernel of 8 × 8 × 8 mm at full-width at half-maximum.

DTI images were preprocessed using FSL 6.0 (FMRIB Software Library, http://www.fmrib.ox.ac.uk/fsl). Diffusion data were corrected for eddy currents and motion using b0 image of each subject. Following brain extraction, individual fractional anisotropy (FA) and mean diffusivity (MD) maps were generated using DTIFIT. The following steps were then processed using the tract-based spatial statistics toolbox in FSL. The FA and MD maps were non-linearly registered to a FMRIB58_FA standard space template, and a mean FA image was created and skeletonized. The mean FA skeleton image was then thresholded at 0.2 to exclude non-white matter voxels, and the FA and MD data of each subject were projected to the mean skeleton.

### Statistical Analyses

For the group comparison of demographic and clinical characteristics of participants, we performed Chi-square test for sex and Kruskal-Wallis test for other variables.

Group comparisons of gray matter and white matter volume were performed in a voxel-wise manner using one-way analysis of covariance (ANCOVA) with age, sex, years of education, and total intracranial volume added as covariates. In addition, correlation analyses of these volumes with diabetes biomarkers (HbA1c and HOMA-IR) in participants with dysglycemia were also performed in a voxel-wise manner using multiple regression analysis with age, sex, years of education, and total intracranial volume added as covariates of no interest. The initial statistical threshold was set at *p*-value of < 0.005 without correction for false discovery rate, and then we used *p*-value of < 0.05 with family wise error correction at cluster level to report the result of statistical significance in the following analyses.

The ANCOVA for group comparisons and the multiple regression for correlation analyses with diabetes biomarkers in participants with dysglycemia were performed by voxel-wise statistics of the FA and MD skeleton maps, using a permutation-based nonparametric test with multiple comparison correction and threshold-free cluster enhancement methods (10,000 permutations). Results were considered significant at *p* < 0.05 corrected for threshold-free cluster enhancement and family-wise error. The potential impact of age, sex, and years of education on the results were minimized by adding these as covariates of no interest.

The gray and white matter volumes from the clusters that showed a significant correlation with diabetes biomarkers in whole-brain correlation analyses were extracted and their correlation with cognitive performance examined in participants with dysglycemia, using Pearson's correlation test.

## Results

Table 1 summarizes the characteristics of the study population disaggregated by study group. There were significant between-group differences with respect to sex distribution and diabetes biomarker levels (FPG, HbA1c, HOMA-IR). There were no significant differences with respect to cognitive performance.

**Table 1:** Demographic and clinical characteristics of subjects^*^.

	**NL** **(***n =*** 14)**	**Prediabetes** **(***n =*** 37)**	**Diabetes** **(***n =*** 23)**	**χ^**2**^**	***p*-value**
**Demographics**					
Female, *n* (%)	11 (78.6)	28 (75.7)	9 (39.1)	9.735	*0.008*
Age, years	72.6 ± 4.2	73.0 ± 6.2	71.3 ± 6.3	0.896	0.639
Education, years	8.4 ± 3.8	10.3 ± 4.7	10.9 ± 3.3	4.043	0.132
**Diabetes biomarkers**					
FPG, mg/dL	92.2 ± 6.3	99.6 ± 8.0	141.8 ± 28.1	46.493	*<0.001*
HbA1c, %	5.4 ± 0.2	5.82 ± 0.2	7.2 ± 0.9	55.843	*<0.001*
HOMA-IR	1.4 ± 0.5	2.1 ± 0.9	3.4 ± 2.0	18.685	*<0.001*
Insulin, μU/mL	6.3 ± 2.0	8.4 ± 3.4	9.6 ± 5.1	4.551	0.103
**Cognitive performance**					
Word list memory	15.1 ± 3.9	15.6 ± 5.0	14.7 ± 5.2	1.402	0.496
Word list recall	3.6 ± 2.8	4.0 ± 2.5	3.0 ± 2.2	2.236	0.327
Word list recognition	8.1 ± 1.9	7.9 ± 2.5	7.2 ± 2.5	3.521	0.172
Constructional recall	4.4 ± 2.9	5.6 ± 2.9	5.4 ± 3.2	1.407	0.495
Constructional praxis	9.3 ± 1.4	9.6 ± 2.2	10.0 ± 1.2	3.027	0.220
Verbal fluency	13.3 ± 3.9	13.2 ± 4.7	12.7 ± 4.8	0.322	0.851
Trail making test A	71.2 ± 30.6	75.0 ± 48.8	59.4 ± 30.3	2.426	0.297
Trail making test B	257.8 ± 108.7	243.4 ± 112.1	227.2 ± 107.6	0.700	0.705
Boston naming test	11.5 ± 2.0	11.5 ± 3.0	11.8 ± 2.5	0.485	0.785

*NL, normal control; FPG, fasting plasma glucose; HbA1c, glycosylated hemoglobin; HOMA-IR, Homeostatic Model Assessment for Insulin Resistance*.

* *Results were obtained using Chi-square test for sex and Kruskal-Wallis test for other variables. All variables other than sex are shown as mean ± standard deviation. Italic values denote statistical significance at the p < 0.05 level*.

**Figure 1:**
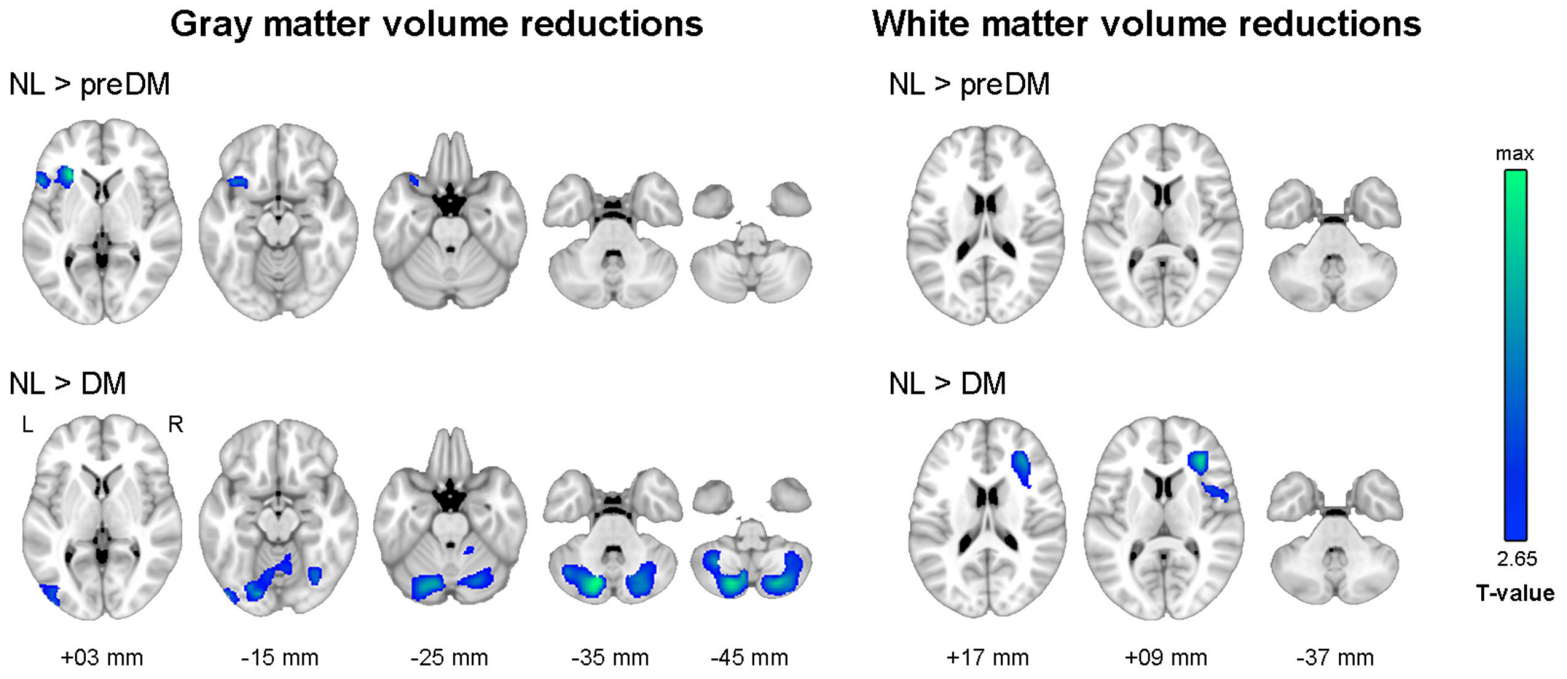
Results of whole-brain analyses showing reduced gray and white matter volume in dysglycemia compared to normal glucose tolerance. Family-wise error corrected *p* < 0.05 at cluster level with underlying voxel level of uncorrected *p* < 0.005. NL, normal control group; preDM, prediabetes group; DM, type 2 diabetes group.

**Figure 2:**
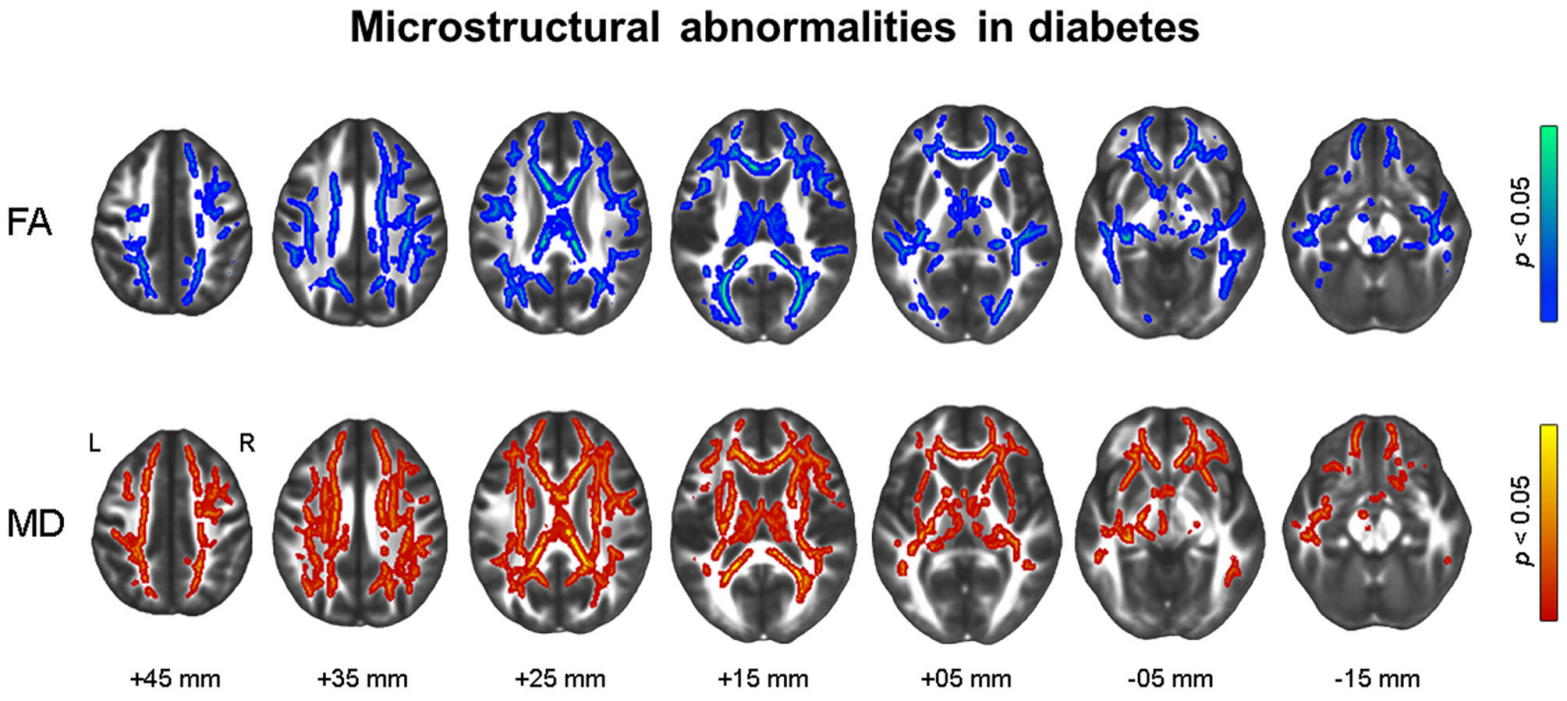
Results of tract-based spatial statistics analysis showing microstructural alterations in diabetes. Threshold-free cluster enhancement and family-wise corrected *p* < 0.05 using nonparametric test. Red color indicates an increase, and blue color indicates a decrease in microstructural measures in diabetes compared to normal glucose tolerance. FA, fractional anisotropy; MD, mean diffusivity.

As illustrated in Figure 1, the diabetes group had smaller gray matter volume in the bilateral cerebellum and occipital lobe and smaller white matter volume in the right prefrontal lobe than the NL group. The prediabetes group had smaller gray matter volume in the left anterior insula and left frontal lobe than the NL group; however, there was no significant difference between prediabetes group and NL group with respect to white matter volume (see Supplementary Table 1). With respect to white matter microstructural alterations, the diabetes group showed widespread reduction in FA and increase in MD compared to the NL group (Figure 2). There was no significant difference in white matter microstructure between prediabetes and NL groups.

**Figure 3:**
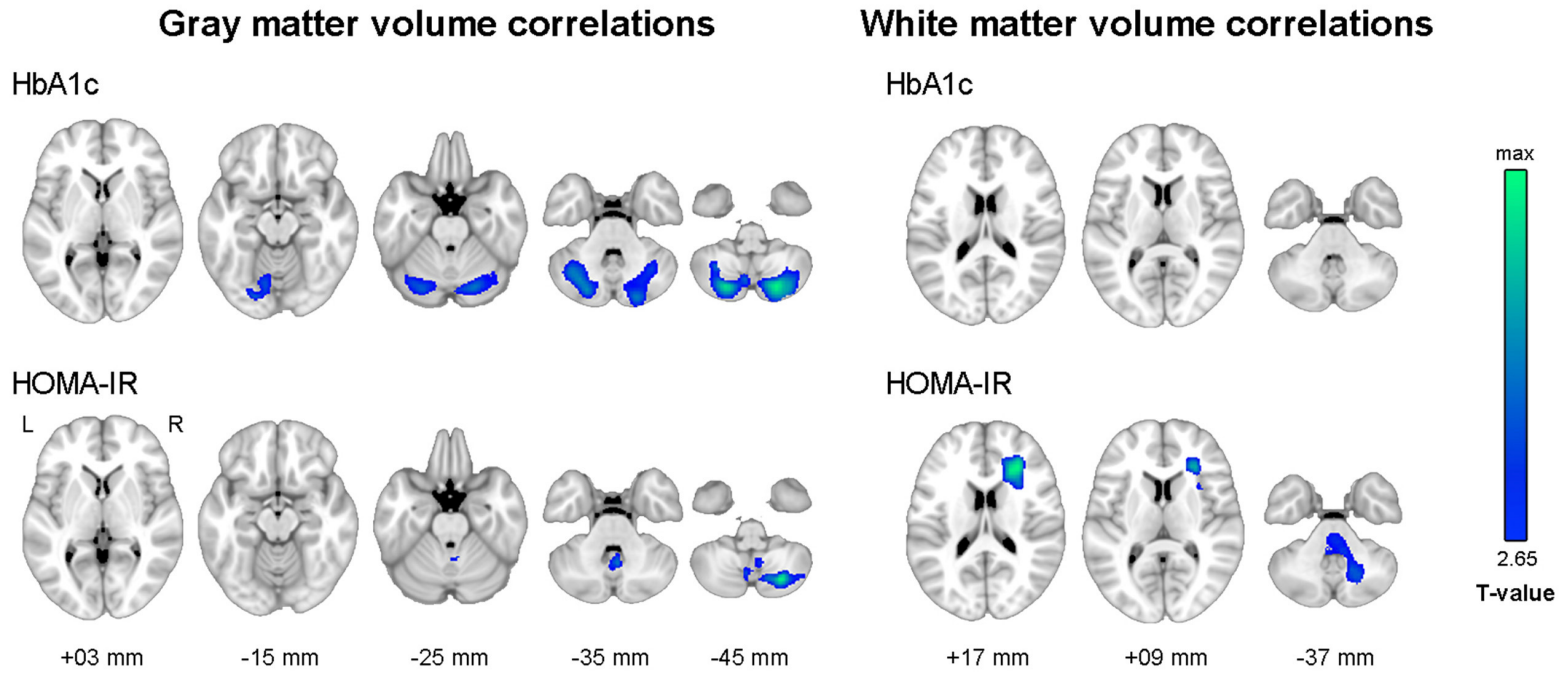
Results of whole-brain correlation analyses between regional brain volume and diabetes biomarkers in dysglycemia. Family-wise error corrected *p* < 0.05 at cluster level with underlying voxel level of uncorrected *p* < 0.005. HbA1c, glycosylated hemoglobin level; HOMA-IR, Homeostatic Model Assessment for Insulin Resistance.

**Figure 4:**
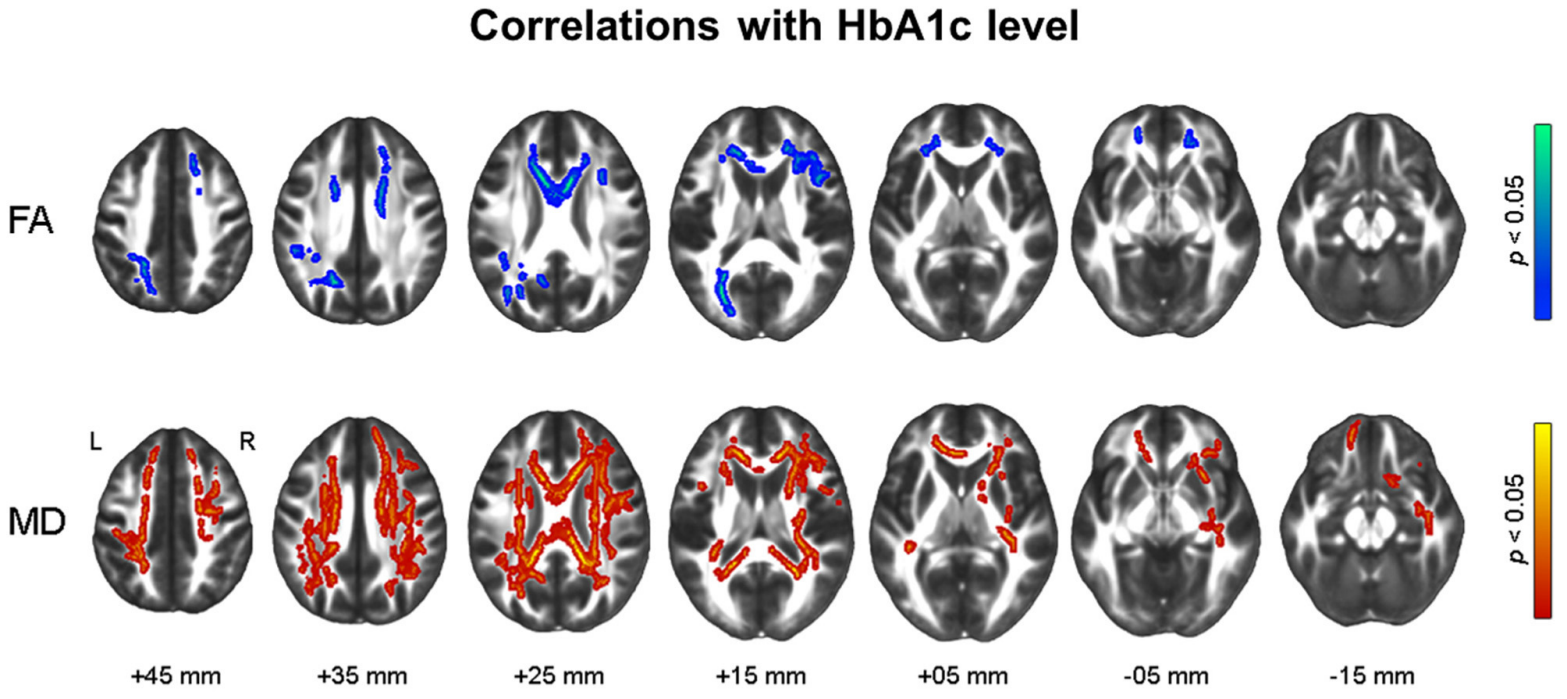
Results of tract-based spatial statistics analysis showing correlations with HbA1c level in dysglycemia. Threshold-free cluster enhancement and family-wise corrected *p* < 0.05 using nonparametric test. Red indicates a positive relationship, and blue a negative relationship of microstructural measures with HbA1c. HbA1c, glycosylated hemoglobin; FA, fractional anisotropy; MD, mean diffusivity.

Figure 3 shows the results of voxel-wise correlation analyses between altered brain volumes and diabetes biomarkers in participants with dysglycemia. HbA1c and HOMA-IR levels showed a negative correlation with cerebellar gray matter volume in participants with dysglycemia. HOMA-IR showed a negative correlation with white matter volume in the right frontal area and left cerebellum (see also Supplementary Table 2). With respect to white matter microstructure, increasing HbA1c level showed a correlation with reduction in frontal FA and more widespread MD in participants with dysglycemia (Figure 4). There was no significant correlation between HOMA-IR level and alteration of white matter microstructure.

Table 2 shows the correlation between cognitive performance and the volume of brain regions which showed a significant correlation with diabetes biomarkers in the preceding analyses. Smaller cerebellar gray matter volume showed a significant correlation with worse performance in the word list recognition test, verbal fluency test, and trail making test B. In particular, the association of cerebellar atrophy with poor performance in word list recognition met the stringent threshold for Bonferroni correction (*p* < 0.005). Smaller white matter volume in the right frontal region showed a significant correlation with worse performance in the word list memory test, word list recognition test, and constructional recall test.

**Table 2:** Correlations between regional volume changes associated with diabetes biomarkers and cognitive performances.

	**GMV in right cerebellum**		**GMV in center cerebellum**		**GMV in right cerebellum**		**WMV in right frontal lobe**		**WMV in center cerebellum**	
	**associated with HbA1c**		**associated with HbA1c**		**associated with HOMA-IR**		**associated with HOMA-IR**		**associated with HOMA-IR**	
	**Correlation**	***p*** **-value**	**Correlation**	***p*** **-value**	**Correlation**	***p*** **-value**	**Correlation**	***p*** **-value**	**Correlation**	***p*** **-value**
Word list memory	0.229	0.059	0.172	0.158	0.178	0.144	**0.260**	**0.031**	0.152	0.213
Word list recall	0.231	0.056	0.222	0.066	0.106	0.385	0.235	0.052	0.109	0.372
Word list recognition	**0.384**	**0.001**	**0.382**	**0.001**	**0.243**	**0.044**	**0.316**	**0.008**	**0.259**	**0.032**
Constructional recall	0.062	0.612	0.119	0.329	0.108	0.378	**0.338**	**0.004**	**0.264**	**0.029**
Constructional praxis	−0.103	0.398	−0.014	0.907	−0.005	0.967	0.044	0.718	0.011	0.930
Verbal fluency	**0.274**	**0.023**	**0.386**	**0.001**	**0.257**	**0.033**	0.201	0.097	**0.253**	**0.036**
Trail making test A	−0.139	0.256	−0.203	0.094	−0.111	0.362	−0.064	0.603	0.000	0.999
Trail making test B	−0.201	0.097	-**0.256**	**0.034**	−0.138	0.259	−0.183	0.133	−0.164	0.178
Boston naming test	0.081	0.509	0.128	0.293	0.033	0.787	0.108	0.378	0.091	0.458

*GMV, gray matter volume; WMV, white matter volume; HbA1c, glycosylated hemoglobin level; HOMA-IR, Homeostatic Model Assessment for Insulin Resistance*.

*Bold values denote statistical significance at the p < 0.05 level*.

## Discussion

Our study examined whole-brain morphological alterations in older adults with dysglycemia. The volume of cerebellar gray matter and frontal white matter, as well as diffuse white matter microstructure, were affected by diabetes, while the insular gray matter was reduced in prediabetes. In particular, structural alterations in cerebellar and frontal areas in dysglycemia showed a correlation with diabetes biomarkers and worse cognitive performance.

To the best of our knowledge, this is the first study to demonstrate a correlation of the levels of hyperglycemia and insulin resistance with cerebellar atrophy in older adults with dysglycemia. We also demonstrated an association of cerebellar atrophy with poor performance in verbal memory recognition and executive function, which is similar to the findings reported in a diabetic middle-aged adult (28). Cerebellum regulates various cognitive functions (such as working memory, executive function, attention, language, and emotional processing) in addition to motor functions (29, 30). Furthermore, cerebellum is one of the brain regions with the most abundant insulin receptors (31). Aberrant insulin receptor-mediated signaling can induce mitochondrial dysfunction, oxidative stress, and proinflammatory cytokine expression, which lead to neural plasticity deficits and atrophic change in the cerebellum (32–34). In addition, impaired glucose homeostasis *per se* alters the expression of cholinergic receptors in the cerebellum, which causes motor and cognitive dysfunction (35). However, none of the previous studies that used region-of-interest analytic methods included cerebellar volume in their analyses (5–22), except for one study which focused only on the motor outcome of diabetes (36). Based on its probable relationship with diabetes biomarkers and cognitive outcomes presented in this study, future research should focus on the structural and functional changes of the cerebellum in dysglycemia.

In line with the previous findings (8, 33, 37–41), this study found reduced frontal white matter volume as well as diffuse white matter dysintegrity involving the frontal area in diabetes. We also found that these structural alterations were affected by hyperglycemia and insulin resistance. Along with cerebellar atrophy discussed above, we speculate that structural changes in the fronto-cerebellar network may be an important neural correlate in diabetic older adults. The cerebellum is connected to the prefrontal cortex and other non-motor cortical regions via projecting fibers and synapses (29). In a recent functional neuroimaging study, patients with type 2 diabetes showed decreased functional connectivity between the cerebellum and dorsolateral prefrontal cortex compared to normal controls (42). Atrophic changes of related structures in diabetes are linked to gait disturbance even in patients without diabetic peripheral neuropathy (36). This study further suggests that impairment of the fronto-cerebellar network may affect not only motor function but also cognitive function, particularly frontal lobe or executive functions which are known to be modulated by feedback mechanisms supported by the cerebellum (29).

Our findings were inconsistent with previous VBM studies (37, 39, 43, 44) that reported a significant difference in the volume of the frontal lobe between diabetes and controls, but not in cerebellar volume. This discrepancy with our results may be attributable to substantial differences in study participants. Unlike the present study, none of the previous studies differentiated their control groups into normal glucose tolerance and prediabetes. Inclusion of a higher proportion of subjects with prediabetes in the control group in previous studies would have obviated the volumetric difference. In addition, the previous studies did not exclude participants with dementia or had used cutoff values of simple screening tests to exclude dementia rather than clinical interview. Furthermore, two of the previous studies (37, 44) included middle-aged adults whose mean age was much lower than those of the current study. As the atrophic change of brain volume is determined by both normal aging and neurodegenerative processes, the differences in age of participants and prevalence of dementia may have contributed to the inconsistent findings compared to the present study.

This study found reduced gray matter volume in anterior insula in prediabetes compared to the NL group, which is consistent with a previous work that reported atrophic changes of the insula and related structures such as cingulate cortex in prediabetes (45). However, we found no significant insular atrophy in diabetes in this study. Insula is known to control interoceptive awareness and executive function against dysglycemia along with the prefrontal cortex, basal ganglia, and cerebellum (46). In patients with type 1 diabetes, chronic dysglycemia was shown to increase functional connectivity of this insula-mediated network to facilitate neuroplastic adaptation to impaired metabolic homeostasis. Furthermore, chronic exposure to dysglycemia decreased cerebellar gray matter volume but increased insular gray matter volume in type 1 diabetes (47). This paradoxical increase of insular volume under chronic dysglycemia may be the result of a neuroinflammatory response to oxidative stress (48) or the compensation for the cerebellar atrophy in diabetes (47). Our results indicate that the alteration of the insula may be more specific to the early stage of dysglycemia, which could be recovered in the later stage of type 2 diabetes.

Our study has strengths over the previous studies. Voxel-based analyses implemented in the current study are automated and observer-independent approaches characterized by accuracy and high sensitivity for identification of local changes (49, 50). The methods allow detection of specific and unexpected structural changes prior to the appearance of clinically relevant symptoms. Additionally, we included a prediabetes group to analyze the structural alterations in the early stage of dysglycemia. However, some limitations of our study should be acknowledged. First, the cross-sectional study design does not permit causal inferences, although we included subjects in the prediabetes and diabetes stage. Second, the small sample size limits the power of the study. Finally, some studies revealed that the disease duration accounts for the brain dysfunction in diabetes. However, information about the disease duration was not available and could not be controlled for in the analyses. Future longitudinal studies with larger sample size are warranted to explore the association between dysglycemia and macro-and micro-structural alterations in the brain.

In summary, the present study using voxel-wise analyses indicates that older adults with dysglycemia may have altered brain structures in the fronto-cerebellar network, and these alterations may reflect diabetes severity and worse cognitive performance. Further longitudinal studies using region-of-interest methods may better clarify the neural correlates involving this network and their cognitive outcomes.

## Data Availability Statement

Data are available on reasonable request to corresponding authors.

## Ethics Statement

The studies involving human participants were reviewed and approved by the Institutional Review Board of the Seoul National University Hospital. The patients/participants provided their written informed consent to participate in this study.

## Author Contributions

DO, J-JJ, SS, YK, and J-YL conceptualized and designed this work. DO, J-JJ, and J-YL drafted the manuscript. All authors were involved in data acquisition and analysis, read, and approved the final version of the manuscript.

## Funding

This work was supported by a clinical research grant-in-aid from the Seoul Metropolitan Government Seoul National University (SMG-SNU), Boramae Medical Center (03-2012-6), and by the National Research Foundation of Korea (NRF) grant funded by the Ministry of Science, ICT and Future Planning (NRF-2014M3C7A1046042), and the Ministry of Education and Science Technology (NRF-2018R1A5A2025964).

## Conflict of Interest

The authors declare that the research was conducted in the absence of any commercial or financial relationships that could be construed as a potential conflict of interest.

## Publisher's Note

All claims expressed in this article are solely those of the authors and do not necessarily represent those of their affiliated organizations, or those of the publisher, the editors and the reviewers. Any product that may be evaluated in this article, or claim that may be made by its manufacturer, is not guaranteed or endorsed by the publisher.
