# Supplementary material for: Brain Structural Alterations, Diabetes Biomarkers, and Cognitive Performance in Older Adults With Dysglycemia
Source: Front Neurol. 2021 Oct 28;12:766216. doi: 10.3389/fneur.2021.766216 (PMC8581483; doi:10.3389/fneur.2021.766216)
Supplement: Supplementary file 1 [file Data_Sheet_1.doc]

**Supplementary table 1. Regions showing reduced volume in dysglycemia compared to normal glucose tolerance**

|  | **Contrast** | **Brain region** | **Cluster size** | **Peak T-value** | **MNI coordinate** | | |
| --- | --- | --- | --- | --- | --- | --- | --- |
|  |  |  |  |  | **x** | **y** | **z** |
| GMV | NL > preDM | Left anterior insula | 2379 | 4.52 | -27 | 24 | 3 |
|  |  |  |  | 4.31 | -33 | 21 | 12 |
|  |  |  |  | 4.03 | -36 | 18 | -20 |
|  | NL > DM | Left cerebellum | 9332 | 5.56 | -15 | -75 | -39 |
|  |  |  |  | 4.29 | -16 | -82 | -14 |
|  |  |  |  | 4.24 | -21 | -90 | -8 |
|  |  | Right cerebellum | 5176 | 4.63 | 32 | -76 | -40 |
|  |  |  |  | 4.59 | 24 | -74 | -42 |
|  |  |  |  | 4.16 | 22 | -72 | -32 |
| WMV | NL > preDM | None |  |  |  |  |  |
|  | NL > DM | Right frontal area | 2320 | 4.67 | 33 | 34 | 12 |
|  |  |  |  | 3.39 | 57 | 0 | 10 |
|  |  |  |  | 3.16 | 40 | 28 | -2 |

GMV, gray matter volume; WMV, white matter volume; NL, normal control; preDM, prediabetes; DM, type 2 diabetes mellitus; MNI, Montreal Neurologic Institute

**Supplementary table 2. Regions showing a negative correlation between tissue volume and diabetes biomarker level**

|  | **Contrast** | **Brain region** | **Cluster size** | **Peak T-value** | **MNI coordinate** | | |
| --- | --- | --- | --- | --- | --- | --- | --- |
|  |  |  |  |  | **x** | **y** | **z** |
| GMV | HbA1c | Right cerebellum | 4819 | 5.00 | 21 | -74 | -48 |
|  |  |  |  | 3.74 | 2 | -75 | -24 |
|  |  |  |  | 3.48 | 9 | -75 | -28 |
|  |  | Left cerebellum | 5125 | 4.46 | -21 | -75 | -42 |
|  |  |  |  | 3.71 | -28 | -69 | -33 |
|  |  |  |  | 3.63 | -32 | -57 | -46 |
|  | HOMA-IR | Right cerebellum | 3149 | 4.17 | 26 | -70 | -50 |
|  |  |  |  | 3.69 | 4 | -52 | -30 |
|  |  |  |  | 3.64 | 33 | -62 | -52 |
| WMV | HbA1c | None |  |  |  |  |  |
|  | HOMA-IR | Right frontal area | 3014 | 3.80 | 24 | 24 | 26 |
|  |  |  |  | 3.74 | 28 | 30 | 16 |
|  |  | Left Cerebellum | 2556 | 3.30 | 16 | -50 | -26 |
|  |  |  |  | 3.24 | 2 | -40 | -21 |

GMV, gray matter volume; WMV, white matter volume; MNI, Montreal Neurologic Institute; HbA1c, glycosylated hemoglobin level; HOMA-IR, Homeostatic Model Assessment for Insulin Resistance
